# Narrative-based medicine in headache disorders

**DOI:** 10.1186/s10194-022-01437-5

**Published:** 2022-06-11

**Authors:** Christian Lampl, Simona Sacco, Paolo Martelletti

**Affiliations:** 1 Department of Neurology and Headache Medical Centre, Konventhospital Barmherzige Brüder Linz, Linz, Austria; 2 Department of Biotechnological and Applied Clinical Sciences, University of L’Aquila, L’Aquila, Italy; 3 Department of Clinical and Molecular Medicine, Sapienza University of Rome, Rome, Italy

**Keywords:** Narrative-based-medicine, Headache care, Patient-centered care, Headache disorders

## Abstract

In this editorial we aim to provide an overview of Narrative-based Medicine (NBM) and highlight what it may offer to the care of individuals with headache disorders.

## Text

In times when different algorithms tend to substitute the decisions taken by physicians and precision medicine is seen as the “promised land” to provide individual care, it is inevitable to see the patients not as a human being but as a numerical product which sums a certain number of processable variables. Sticking only to variables and forgetting the importance of human-to-human interaction represent a new challenge modern medicine is offering. In this editorial we aim to provide an overview of Narrative-based Medicine (NBM) and highlight what it may offer to the care of individuals with headache disorders.

The correct clinical diagnosis of headache disorders is pursued by a comprehensive acquisition of patients´ characteristics, general medical status, headache features with associated symptoms, frequency and intensity of the headache episode(s), examinations reports, and pharmacological history [1]. An appropriate headache management includes a cooperative relationship with the patients, including a attentive understanding of their needs. Traditionally the model of care has been disease-centered, slowly evolving in past years to a patient-centered care model (PCC), where the privileged approach is individualized for each patient [2], together with an evidence-based system providing the sound scientific basis for the treatment.

PCC is a well-accepted model in general clinical practice [3]; it includes the bio-psycho-social aspects of the disease and its holistic care. However, in order to value different points of view of both the doctor and the patient [4] and offer emotional reward to both parties, another tool needs to be implemented, NBM, which applies narrative ideas to the practice of medicine [5]. It is particular important to promote the NBM approach because while Evidence-Based Medicine (EBM) was imposing itself as the ruling model, narratives were gradually substituted by data on symptoms and diseases, considered to be more scientific. Rita Charon and John Launer, two of NBM´s key proponents, pointed out the importance of narratives in healthcare and how a narrative competence is required in order to understand and be touched by the stories around the illnesses of the patients [6, 7]. Both parties of the therapeutic process have to tell a story: the patients tell us about their headache episodes, the symptoms, the pain they feel and how it impacts their lives. Moreover, they tell us about their fears, their possible explanations of their headache, what they did so far without experiencing sufficient benefit, and what they wish for their future. On the other side, the headache specialists come up with their experience, formulate a possible diagnosis, explains the nature of the disease, its triggers and aggravating factors, the course over life, and illustrate the treatment options. Both stories are told based on interindividual personalities, knowledge, and experiences. If the physician does not show interest for the patient and gives no empathy for their condition, the patient may not tell the right story and omit emotionally important aspects which deserves care. This may lead to unnecessary examinations and consultations.

During the coversation lots of conscious or unconscious personal interactions are observable. Patients come to the consultation with many variable expectation: “Will the headache specialist listen to me? Is there an interest in my story? Can I tell my story in detail? Will they address my concerns? What will the headache specialist advise me? Will the specialist provide a good solution for my pain?” The headache specialist, on the other hand, might feel uncomfortable while listening to difficult patients, or feeling the pressure of a great number of patients, incorporating co-morbidities and multiple psychosocial effects.

The NBM approach aims to take personal narrative styles into account [8], recognizing narrative skills as a central part to the practice [9]. In NBM, description of experiences is an important part of the consultation. The narrative includes not only words, but also silences, physical reactions, gestures. This approach provides a richer experience of patient perspective and needs. While the physicians listen and observe the patients, they understand the patient and learn valued and devalued issues. Good empathy and communication skills are fundamental to any consultation [10, 11]. Those are not commonly teached at University and mostly learned on the field. Additionaly, they also rely on the physician background, personality and attitude and even in the same physician they are subject to variations due to emotional status, tiredness, time and physical contraints, personal feelings. In order to apply NBM during the consultation, John Launer identifies 7 principles—the 7 *C*s [12]. *Conversation*, *Curiosity*, *Context*, *Complexity*, *Challenge, Caution*, *Care* [13]. Throughout these 7 Cs, adherence should be increased. To increase this fundamental need of adherence Rita Chanon defined 4 divides [14]: the “*context of illness”* (a biological phenomenon requiring medical intervention), “*beliefs about disease causality”* (that often differs between the headache specialist and the patient), as well as “*shame, blame and fear*” (headache makes them vulnerable and fearful) and the “*relation to mortality”* (illness as an unexpected event). The foundational models of NBM are crucial in dealing with disparities in headache care due to racial, ethnic, and socioeconomic status. Every clinical decision should be guided by the needs and the values of both parties in the treatment process. NBM allows headache patients to unburden themselves. To achieve that, the headache specialist must focus on the need to understand, rather than the need to solve the headache problem. Active attentive listening is intrinsically therapeutic [15]. Using a narrative approach in daily clinical practice means being open towards patients and their narratives through the use of specific narrative skills such as:

- Understanding the patients and their experience with the illness;
- The diagnosis must be established and explained in an individual context, avoiding any systematic and standard description of the disease;
- Use of communication skills, such as exploring, conjecturing, planning, active listening and circular questioning [16];

- Self-reflection.

*“By recognizing that the language of medicine and the language of the patient's world transformed by illness are not the same, the medical humanist creates a communication bridge. And in so doing, provides support to both doctor and patient as they face uncertainty”* [17].

Migraine is the third highest cause of disability worldwide [18], it affects people of all ages, races and ethnicity [19]. There is a wide evidence that effectively treating migraine reduces the burden of the disease and the risk of medication overuse. Individuals with primary headaches need physicians who understand their disease, provide a clear diagnosis and explanations to symptoms, can reassure them on the nature of their symptoms, can prescribe the most appropriate treatment, and accompany them throughout their illness. NBM might be a very useful tool in the clinical practice of primary headaches. This is particularly true when the sole medical storytelling is the pillar for a correct diagnosis, in absence of reliable biomarkers, as is the case of all primary headaches. It is important that both the headache specialist and the patient are open for a narrative exploration and the headache specialist is trained in communication skills. Headache specialists lean toward a not-perfect biomedical model of e.g. migraine, following an EBM approach of treatment possibilities. But there may be a lack of care for not taking into account migraine patients’ apprehensions and needs, concentrating only on the numerical data on headache days, analgesics intake, disability scores. Co-morbidity is increasing and becomes more prevalent and EBM does not work well in multiple conditions and in giving a complex bidirectional clinical lectures, as for example in the relationship migraine/depression [20]. While PCC is found to be especially effective in managing co-morbidites [21], NBM is also relevant since listening to patient’s narratives is beneficial for both doctor and patient [22] and it helps building a strong relationship. Narratives could then fill the gap between the large amount of data coming from randomized-controlled studies and the ability of the physicians to apply this knowledge to a single case. Therefore, EBM and NBM should be applied in complementary terms [23]. EBM alone cannot help patients in their emotional aspects. Physicians have to improve their skills in recognizing the plights of the individuals they are caring, to extend empathy toward them and to be honest in the not uncommon situations were the disease is difficult-to-treat and refractory to medical treatments [24]. NBM can offer better rewards to physicians and health care providers in general by enhancing human interactions and having patients more satisfied and emotionally-closed. Indeed application of NBM may be a powerful therapeutic instrument itself and favor patient engagement, compliance by also favoring any placebo effect of treatments and reducing the nocebo effect. The hypothesis to be tested in primary headache care is that physicians equipped with the skills for a NBM approach achieve better outcomes in managing patients than those not equipped with a NBM approach.

In order to pursue a NBM approach, we must also take into account the current rules in the National Health Systems regarding time slots for the length of a visit, the enormity of the patients who require headache consultations and the consequent formation of waiting lists that creates disparities and favors chronicization and self -medication overuse, and cause patients’ expectations to grow dramatically. These barriers must be filled also by active and exstensiv involvement of a multidisciplinary team with different expertise which may help to allocate enough time for a NBM approach. This approach is fundamental in an epidemiological context that tends to see headaches grow globally [25] and which must appropriately and non-exclusively use models for assessing disability in the various personal, social and occupational domains [26].

Adequate mutual listening between doctor and patient, not just unidirectional, can reinforce therapeutic adherence [27], increase the physicians’ capacity for deprescription [28], favor a multidisciplinary intervention that is still experienced as a mere act of delegation [29] and facilitate telehealthcare contacts to maintain the continuity of a personalized relationship [30, 31].

## Data Availability

Not applicable.
